# The Role of Mcl-1 in *S. aureus*-Induced Cytoprotection of Infected Macrophages

**DOI:** 10.1155/2013/427021

**Published:** 2013-01-28

**Authors:** Joanna Koziel, Katarzyna Kmiecik, Daniela Chmiest, Katarzyna Maresz, Danuta Mizgalska, Agnieszka Maciag-Gudowska, Piotr Mydel, Jan Potempa

**Affiliations:** ^1^Department of Microbiology, Faculty of Biochemistry, Biophysics and Biotechnology, Jagiellonian University, Ul. Gronostajowa 7, 30-387 Kraków, Poland; ^2^Broegelmann Research Laboratory, The Gade Institute, University of Bergen, 50021 Bergen, Norway; ^3^Oral Health and Systemic Diseases Research Group, School of Dentistry, University of Louisville, Louisville, KY 40204, USA

## Abstract

As a facultative intracellular pathogen, *Staphylococcus aureus* invades macrophages and then promotes the cytoprotection of infected cells thus stabilizing safe niche for silent persistence. This process occurs through the upregulation of crucial antiapoptotic genes, in particular, *myeloid cell leukemia-1 (MCL-1)*. Here, we investigated the underlying mechanism and signal transduction pathways leading to increased *MCL-1* expression in infected macrophages. Live *S. aureus* not only stimulated *de novo* synthesis of Mcl-1, but also prolonged the stability of this antiapoptotic protein. Consistent with this, we proved a crucial role of Mcl-1 in *S. aureus*-induced cytoprotection, since silencing of *MCL1* by siRNA profoundly reversed the cytoprotection of infected cells leading to apoptosis. Increased *MCL1* expression in infected cells was associated with enhanced NF*κ*B activation and subsequent IL-6 secretion, since the inhibition of both NF*κ*B and IL-6 signalling pathways abrogated Mcl-1 induction and cytoprotection. Finally, we confirmed our observation *in vivo* in murine model of septic arthritis showing the association between the severity of arthritis and Mcl-1 expression. Therefore, we propose that *S. aureus* is hijacking the Mcl-1-dependent inhibition of apoptosis to prevent the elimination of infected host cells, thus allowing the intracellular persistence of the pathogen, its dissemination by infected macrophages, and the progression of staphylococci diseases.

## 1. Introduction

*Staphylococcus aureus* is a major cause of community-acquired and nosocomial infections, including localised and systemic life-threatening conditions, such as osteomyelitis, endocarditis, pneumonia, and septicaemia [1]. Despite the increasing morbidity and mortality due to staphylococcal infections, relatively little is known about the molecular mechanisms by which this pathogen disseminates systemically. Recent studies have shown that *S. aureus* not only survives phagocytosis by neutrophils and macrophages but is also able to persist inside these cells [2, 3]. As is the case for *Listeria monocytogenes* and *Mycobacterium tuberculosis* [4–6], long-term survival, especially inside macrophages, may be a mechanism of dissemination of staphylococci. This hypothesis is further supported by the observation that intracellular *S. aureus *manipulates macrophage cell signalling processes and transcription to promote survival of infected phagocytes without bacterial eradication [7]. Therefore, precise elucidation of the mechanism of induction of cytoprotection in macrophages, a potential vehicle for pathogen spreading in the host, is of high importance.

Recently, we showed that live intracellular bacteria induce cytoprotection in human and murine macrophages, allowing the pathogen to silently persist and remain invisible to the immune system [3, 7]. We proposed that the pathogen induces a prosurvival signalling pathway through the induction of expression of antiapoptotic factors, including myeloid cell leukemia-1 (Mcl-1), an antiapoptotic protein of the Bcl-2 family. Mcl-1 possesses BCL-2 homology (BH) domains 1–4, similar to other antiapoptotic BCL-2 family members (BCL-2, BCL-XL, BCL-W, and A1). It inhibits cell death by sequestering proapoptotic proteins (e.g., BAX, BAK, BIM, PUMA, and BAD), thus stabilising the mitochondrial membrane and preventing release of cytochrome *c *[8, 9]. *MCL1* expression can be induced by survival and differentiation signals such as cytokines and growth factors produced as a result of activation of a number of well-known signal transduction pathways (e.g., MAP kinases, PI3K/Akt, JAK/STAT, and NF*κ*B) [10]. Cellular levels of Mcl-1 are regulated also by alteration of this protein turnover rate, which is dependent on PEST sequences within the N-terminal region of Mcl-1 and other motifs that target the protein for degradation by the proteasome [8].

In the current paper, we documented the elevated level of Mcl-1 in *S. aureus*-infected human primary macrophages and joints in murine model of septic arthritis, associated with infiltration of macrophages into the synovia. We showed for the first time that small inhibitory (si)RNA silencing of *MCL1* in *S. aureus*-infected macrophages abrogates cytoprotection against staurosporine-induced apoptosis, confirming the role of Mcl-1 in sustaining the viability of macrophages during infection. Furthermore, we established that *S. aureus* induced Mcl-1 through signalling pathways that required NF*κ*B and IL-6, since inhibition of these pathways partly suppressed bacterial-mediated enhancement of Mcl-1 expression and cytoprotection. Therefore, we propose a model in which expression of prosurvival genes, such as *MCL1, *enables virulent *S. aureus* strains to circumvent cell death, thus ensuring a safe ecological niche prior to dissemination. Regulation of human macrophage longevity by intracellular *S. aureus* has clinical relevance for understanding the pathogenesis of *Staphylococci*-caused infectious diseases.

## 2. Materials and Methods

### 2.1. Cell Culture

PBMCs were isolated from human blood using a Lymphocyte Separation Medium (PAA) density gradient yielding the fraction highly enriched in monocytes (90% CD14-positive) as described previously [7]. Cells were plated at 3 × 10^6^/well in 24-well plates (Sarstedt) in RPMI1640 (PAA) supplemented with 2 mM L-glutamine, 50 *μ*g/mL gentamicin (Sigma), and 10% autological human serum. After 24 h, nonadherent PBMCs were removed by washing with complete medium, and adherent cells were differentiated to hMDMs in this medium for 7 days with fresh medium changed every 2 days. Blood was obtained from the Red Cross, Krakow, Poland. The Red Cross de-identified blood materials as appropriate for human subjects confidentiality assurance. Thus, the current paper adheres to appropriate exclusions from human subjects approval. The murine macrophage cell line RAW 264.7 obtained from American Type Culture Collection was maintained in DMEM (PAA) supplemented with 5% fetal bovine serum. 

### 2.2. Bacterial Strains, Storage, and Growth Conditions

The laboratory *S. aureus* strain Newman was kindly provided by Dr. T. Foster (Trinity College, Dublin, Ireland) and *E. coli *strain was from laboratory stocks. Bacteria were grown to the stationary growth phase at 37°C overnight under constant rotation (180 rpm), then were collected by centrifugation (5,000 ×g, 8 min), washed with phosphate-buffered saline, and resuspended in PBS to the desired OD_600 nm_. Staphylococci were opsonized by incubation at 37°C for 30 min in heat inactivated FCS, washed, and resuspended in PBS. Numbers of vital bacteria in samples used in phagocytosis assay were routinely verified by plating dilutions on agar plates and counting colonies to determine CFU per mL. Heat treatment (80°C for one hour) was used to kill bacteria.

### 2.3. Macrophages Infection

Macrophage infection was performed using *S. aureus* and *E. coli *as described previously [7]. Unspecific cell activation by phagocytosis of inert particles was determined using latex beads (1.1 *μ*m; Sigma) as described previously [7]. Phagocytosis assays were carried out for 2 hours at 37°C at a multiplicity of infection (MOI) of 1 : 50 (hMDMs) or 1 : 5 (RAW 264.7), resulting in >85% of macrophages engulfing at least one bacterium. After that time cells were rinsed 4 times with ice-cold phosphate-buffered saline. Any remaining nonphagocytosed bacteria were killed by culturing in medium containing gentamicin (50 *μ*g/mL) for 24 h. The medium was then replaced with fresh media without antibiotics, and cultures were maintained for the desired time. Cells treated identically, but without bacteria, were analyzed in all experiments as a control (mock-infected cells).

For inhibitor experiments, before inoculation with bacteria or applying control stimulus, macrophages were preincubated for 30 min with nontoxic doses (data not shown) of the NF*κ*B inhibitor Bay 11-7082 (2–40 *μ*M). Where indicated, cycloheximide (a final concentration 10 *μ*g/mL) was added 30 min before infection with bacteria and present in media until cells were washed 30 min after infection. This treatment reduced *de novo* translation of proteins in macrophage by 95.5% as determined by ^35^S-Met incorporation (data not shown). 

### 2.4. Viability Assays

After *S. aureus* phagocytosis and/or treatment with compounds inducing apoptosis, macrophage viability was examined by lactate dehydrogenase (LDH) release. The LDH release assay was performed using a CytoTox96 Non-Radioactive Lactate Dehydrogenase Cytotoxicity Assay kit (Promega). Infected and control hMDMs or RAW 264.7 cells in a 24-well tissue culture plate (3 × 10^5^ cells per well) were treated with 1 *μ*M staurosporine (STS; Sigma) added 2 h or 24 h after-infection as a stimulator of apoptosis. Samples were then incubated for 6 h or 24 h, and 200 *μ*L of culture medium were withdrawn and subjected to analysis as described previously [7].

### 2.5. Analysis of Caspase-3 Activity

The activity of caspase-3 was determined by release of 7-amino-4-trifluoromethyl-coumarin (AFC) from a DEVD-AFC peptide substrate. Cells, both control and exposed to *S. aureus*, with or without apoptotic stimuli, were collected by centrifugation (200 ×g, 5 min, 4°C), washed with ice-cold PBS, and resuspended in 100 *μ*L of lysis buffer (50 mM Tris, pH 7.5, 150 mM NaCl, 1% NP-40, 0.5% deoxycholic acid, and 0.1% SDS). Samples were then incubated on ice for 20 min and subjected to centrifugation (16,000 ×g, 10 min). The protein content of supernatants was measured using a BCA method, and caspase activity was determined by using a Spectra Max Gemini EM (Molecular Devices) as described previously [7]. 

### 2.6. Protein Isolation and Immunoblotting

Whole cellular extracts from control and stimulated cells were prepared using 100 *μ*L of RIPA-lysis buffer (0.25% Na-deoxycholate, 0.5% Nonidet P-40, 0.05% SDS, protease inhibitor cocktail, and 2.5 mM EDTA in PBS) and stored at −20°C. The joints of DBA1 mice were homogenized in 300 *μ*L of RIPA-lysis buffer. Equal amounts of protein (40 *μ*g/well) were separated using SDS-PAGE (12% or 16% gels depending on molecular mass of proteins of interest) and electrotransferred onto nitrocellulose membranes (BioRad) in buffer composed of 25 mM Tris, 0.2 M glycine, and 20% methanol (30 V, overnight). Nonspecific binding sites were blocked with 3% BSA in TTBS buffer (20 mM Tris, 0.5 M NaCl, and pH 7.5 with 0.05% Tween 20) for 1 h, followed by 1-2-hour incubation with the relevant primary antibody: 100-fold diluted anti-Mcl-1 (Santa Cruz), or 3,000-fold diluted anti-*β*-actin (Sigma). Membranes were washed extensively in TTBS buffer and incubated with secondary horseradish peroxidase-(HRP-) conjugated antibodies, 10,000-fold diluted donkey anti-rabbit IgG, or 20,000-fold diluted sheep anti-mouse IgG, for 1 h in TTBS buffer containing 1% BSA. Membranes were washed (4 × 15 min) in TTBS buffer, and blots were developed using ECL detection (Western Blotting Detection Reagents; Amersham Biosciences).

### 2.7. Densitometric Analyses

Densitometric analyses of western blots were performed using Kodak Digital Software. Results are presented as mean values of arbitrary densitometric units corrected for background intensity or as the fold of increase over a level characteristic for nonstimulated cells. 

### 2.8. Quantitative PCR (qRT-PCR)

Total cellular RNA was extracted from cultured hMDMs using aRNeasy Mini Kit (Qiagen) according to the manufacturer's instructions. RNA samples were DNase treated, and cDNA was prepared by reverse transcription using RevertAidTM First Strand cDNA Synthesis Kit (Fermentas). Five hundred nanograms of RNA from each sample were used for cDNA synthesis reaction with oligo(dT) primers according to the manufacturer's instructions. Quantitative PCR reaction was performed with an SYBR Green method in a reaction volume of 20 *μ*L, containing 1 *μ*L of cDNA sample, 0.5 *μ*M of each primer, and 1x SYBR Green JumpStart Taq Ready Mix (Sigma). qRT-PCR forward and reverse primers for *IL-6*, *MCL1*, and *MCL1S* genes and for the housekeeping *EF-2* gene (used for normalization) are listed in Table 1. After 5 min of initial denaturation at 95°C, reactions were carried out for 40 cycles at the given conditions: denaturation, 95°C, 20 sec; annealing, 56°C–62°C (as shown in Table 1), 60 sec; extension, 72°C, 60 sec; followed by a final elongation step at 72°C for 10 min. All the reactions were performed in duplicates. Means for threshold cycle (Ct) values were calculated and analyzed using the “delta-delta Ct” quantification method [11]. Routinely, for the evaluation of quality of qRT-PCR reactions, samples were resolved on nondenaturing 1.5% agarose gels and visualized by staining with ethidium bromide.

### 2.9. Cytokine Assay

Two hundred *μ*L of cell culture supernatants were collected and stored at −80°C until analysis. The level of IL-6 was determined by using commercially available ELISA kits according to the manufacturer's instructions (R&D Systems).

### 2.10. Transfection with siRNA

Silencing of the *MCL1 *gene expression was accomplished by transfection of cells with specific siRNA or with negative control siRNA (Accell Smart pool and Control pool, resp., Thermo Scientific Dharmacon). Briefly, for each of transfected wells, siRNA (final concentration 60 nM) was combined with 3 *μ*L of lipofectamine 2000 (Invitrogen) in Opti-MEM medium (Invitrogen). Transfection was performed for 4 h followed by 24 h of normal cell growth before desired assays were performed.

### 2.11. Evaluation of NF*κ*B Activity

NF*κ*B activity was measured in nuclear protein extracts by the EMSA method. Nuclear extracts were prepared by a miniextraction procedure as described before [12]. The protein concentration was measured with bicinchoninic acid (BCA method), and the obtained extracts were frozen (−80°C) in 10% glycerol. For the NF-*κ*B-directed EMSA, double-stranded probes were prepared using the pair of primers AGCTTCAGAGGGGACTTTCCGAGAGG and AGTCTCCCCTGAAAGGCTCTCCTCGA. EMSA was performed as described before [12].

### 2.12. Septic Arthritis Induction and Examination of Infection

Male DBA1 mice (8–12 weeks old) were obtained from the breeding unit of the Department of Human Developmental Biology, Jagiellonian University, School of Medicine. Mice were fed autoclaved food and water. All experiments were conducted according to guidelines of the Animal Use and Care Committee of the Jagiellonian University School of Medicine. Mice were inoculated with *S. aureus* in the tail vein (5 × 10^7^ in 200 *μ*L) at day 0. The overall condition was evaluated by assessment of body weight and general appearance. The hind paws and forepaws were inspected every second day. Animals were observed daily for the presence of arthritis, and the clinical severity of disease was scored for each paw on a scale of 0–4, with the index being the sum of the scores for all four paws. The criteria for the grading were as follows: 0: no evidence of erythema and swelling; 1: mild erythema and swelling of the wrist or the ankle; 2: moderate erythema and swelling from the wrist to the metacarpal joints or from the ankle to the metatarsal joints; 3: severe erythema and swelling of the entire paw including digits; 4: maximal erythema and swelling of the paw. The bacterial load in joints, kidney, and spleen was examined at time of sacrifice (8 day p.i.). The organ homogenates were plated on tryptic soy agar and CFU counted after overnight incubation at 37°C.

### 2.13. Statistics

Results were analyzed for statistical significance using the nonparametric Student's *t*-test. Differences were considered significant when *P* < 0.05.

## 3. Results

### 3.1. *S. aureus* Specifically Induces Mcl-1 Expression in Human Macrophages

**Figure 1:**
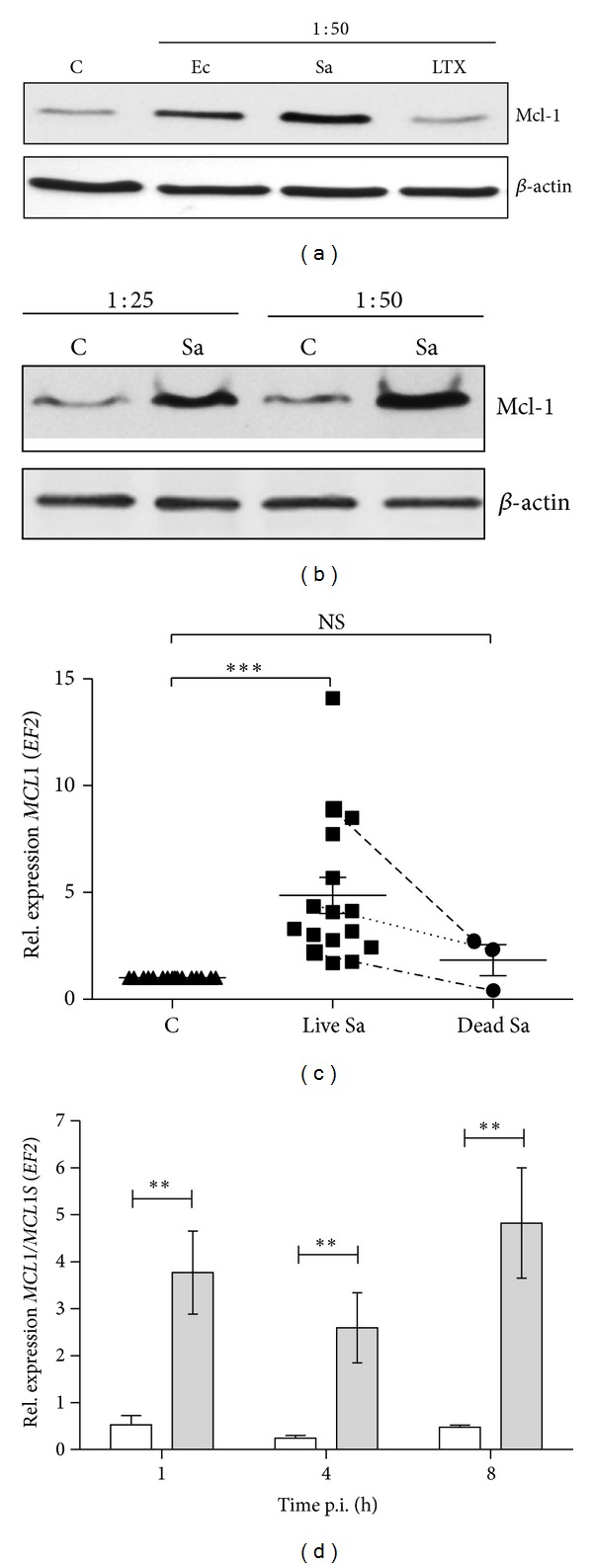
Mcl-1 expression is induced specifically by *S. aureus *phagocytosis and is dependent on bacterial dose and viability. (a) The effect of phagocytosis of *S. aureus *(Sa), *E. coli* (Ec), and latex beads (LTX) on Mcl-1 protein levels was assessed by immunoblot. After phagocytosis, cells were cultured for 8 h and then protein fractions were prepared as described in Section 2. Representative immunoblot from three separate experiments performed on hMDMs derived from different donors is shown. Mcl-1 was visualised by immunoblot using anti-Mcl-1-specific antibodies. (b) The effect of *S. aureus* phagocytosis on Mcl-1 levels is proportional to an infection dose. A representative immunoblot from three separate experiments performed on hMDMs derived from different donors is shown. (c) The effect of phagocytosis of live (Sa) and dead, heat-killed *S. aureus* (Sa HI) on *MCL1* expression was determined by qRT-PCR. Data are from independent reactions using hMDMs derived from different donors. Paired points represent the response of hMDMs obtained from the same donors to both live and dead bacteria. Bars represent relative means ± SD. ***P < 0.001; NS: not significant. (d) The comparison of expression levels of proapoptotic *MCL1S* (white bars) versus antiapoptotic *MCL1 *(filled bars). Results were obtained by qRT-PCR from three separate experiments. **P < 0.01.

We previously showed that *S. aureus *can protect infected macrophages against apoptosis through upregulation of expression of antiapoptotic genes [7]. Among these genes, *MCL1* plays a key role in macrophage survival [13, 14]. Here, we investigated potential mechanisms of Mcl-1 regulation, as well as its role in cytoprotection induced by *S. aureus*. The Mcl-1 induction in response to phagocytosis of different bacteria and particles was examined by incubating macrophages with *S. aureus*, *E. coli,* or latex beads for 8 h. *S. aureus* induced the highest level of Mcl-1, about five-times more (4.78 ± 0.96-fold above the control level) than that seen in mock-infected cells (Figure 1(a)). By contrast, no change in Mcl-1 levels was observed after incubation with latex beads (1.06 ± 0.06) and only a slight upshift after *E. coli* (1.78 ± 0.63) (Figure 1(a)). The enhanced Mcl-1 production in response to *S. aureus *was proportional to infection rate (Figure 1(b)) and was exerted only by viable bacterial cells (Figure 1(c)). The latter was clearly apparent from a comparison of relative levels of *MCL1* mRNA induced in response to live or dead bacteria in macrophages derived from different blood donors (8.87 versus 2.75, 4.36 versus 2.35, and 2.24 versus 0.4, resp.). An intriguing feature of *S. aureus*-induced Mcl-1 expression was the synthesis of an alternatively spliced *MCL1 *gene product (*MCL1S* proapoptotic isoform), which was observed at the mRNA level early after infection (Figure 1(d)). Significantly, however, the expression of *MCL1S* was at much lower level compared to the full-length, antiapoptotic *MCL1 *form (Figure 1(d)). 

Taken together, these results suggested that stimulation of Mcl-1 expression in macrophages is preferentially induced by viable *S. aureus.*

### 3.2. *S. aureus *Influences Mcl-1 Synthesis and Turnover Rate

**Figure 2:**
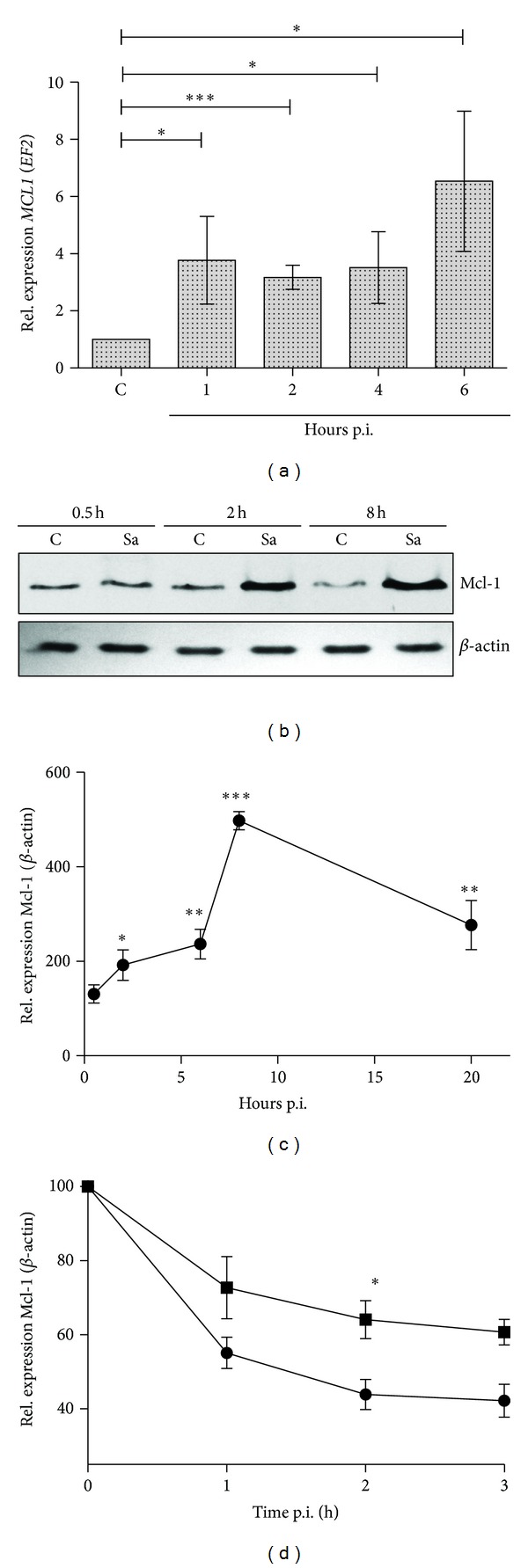
*S. aureus* increases both *de novo *Mcl-1 synthesis and stability. (a) Time course of *MCL1* expression in control and *S. aureus*-infected macrophages (hours after-infection; p.i.) was monitored by qRT-PCR, as described in Section 2. Data represent the mean values calculated from the results of three independent experiments using hMDMs derived from different donors. Bars represent mean relative expression ± SD. *P < 0.05; ***P < 0.001. (b, c) Time course of Mcl-1 protein synthesis following *S. aureus* infection. Mcl-1 levels were measured at different time points between 0.5 and 20 h p.i. by immunoblot. (b) Representative immunoblot from three separate experiments performed on macrophages derived from different donors. (c) Relative Mcl-1 levels obtained by densitometric analyses of western blots. Results from three separate experiments. Data represent means ± SD. *P < 0.05; **P < 0.01; ***P < 0.001. (d) Mcl-1 stability in macrophages incubated in the presence of cycloheximide (CHX) (10 *μ*g/mL) in the absence (circles) or presence (squares) of *S. aureus* (MOI 1 : 50). At time periods of up to 3 h, Mcl-1 levels were detected by immunoblot in cell lysates. Data represents Mcl-1 levels relative to time 0, which was arbitrarily set as 100%, obtained by densitometric analyses of western blots. Data represent means ± SD of three separate experiments. *P < 0.05.

To correlate *S. aureus*-induced cytoprotection with the expression of *MCL1,* we determined the time dependence of the induction of specific mRNA after macrophage challenge with bacteria. A 4-fold increase of *MCL1* mRNA was observed 1 h after-infection, with sustained upregulation observed for up to 6 h (Figure 2(a)). At the protein level, Mcl-1 levels were significantly increased 2 h after-infection, reached a maximum at 8 h, and remained at 3-fold higher levels compared to mock-infected cells for at least 20 h (Figures 2(b) and 2(c)).

Since the observed high levels of expression of Mcl-1 in *S. aureus*-infected macrophages could be the result of either *de novo* synthesis or the decreased turnover rate, we compared Mcl-1 stability in macrophages treated with cycloheximide in the absence and presence of *S. aureus*. As shown in Figure 2(d), in mock-infected cells, blocking *de novo *biosynthesis resulted in a rapid decrease in the cellular levels of Mcl-1. By contrast, in cells infected with *S. aureus*, the level of Mcl-1 was significantly higher (Figure 2(d)). Cumulatively, these results clearly showed the dual nature of the effects of *S. aureus* on Mcl-1, which involved increased Mcl-1 protein synthesis as well as increased protein stability.

### 3.3. The Mcl-1 Expression Correlates with Prevalence and Severity of *S. aureus*-Induced Arthritis

**Figure 3:**
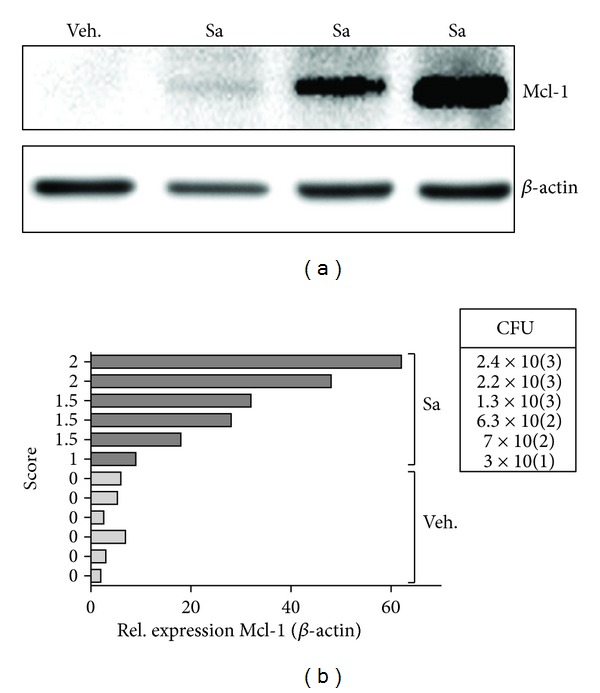
Exposure to *S. aureus* triggers Mcl-1 production *in vivo.* (a) Immunoblot reveals increase in Mcl-1 expression in inflamed joints derived from three individual *S. aureus*-infected mice (Sa) in comparison to noninfected animals (Veh.). A representative immunoblot from three separate experiments is shown. (b) The expression of Mcl-1 in joints correlates with infection score and bacterial load determined as described in Section 2. Data represents Mcl-1 levels in noninfected (Veh.; *n* = 6) and *S. aureus*-infected (Sa; *n* = 6) animals, obtained by densitometric analyses of western blots.

*S. aureus *is the causative agent in about 60% of nongonococcal bacterial arthritis cases, a disease characterized among others by robust influx of macrophages and their sustain activation in joints [15, 16]. Therefore, we determined Mcl-1 expression in inflamed joints in the previously established murine model of *S. aureus* arthritis [17]. To this end DBA1 mice were injected i.v. with 5 × 10^7^ CFU, a dose causing a low mortality rate (see Supplementary Figure 1(a) in Supplementary Material available online at http://dx.doi.org/10.1155/2013/427021). At day 8 after injection all animals showed clear symptoms of arthritis (Supplementary Figure 1(b)). Bacteriological examination of joints, spleen, and kidneys revealed the abundant load of *S. aureus *in 100% of mice (Supplementary Figure 1(c)). This finding correlates with inflammatory response manifested by IL-6 secretion (Supplementary Figure 1(d)) and splenomegaly (data not shown). Further investigation of the relationship between both clinical and bacteriological signs of arthritis and Mcl-1 expression in joints revealed the significant association (Figures 3(a) and 3(b)). We found Mcl-1 expression being significantly upregulated in *S. aureus*-positive joints (32.83 ± 7.94-fold above the control level) in comparison to noninfected tissues (4.29 ± 0.82-fold above the control level, *P* < 0.001). Furthermore, we also observed positive association between the Mcl-1 expression level and bacterial load in joints. This indicates that *S. aureus* induced Mcl-1 expression also *in vivo *in the inflamed tissue.

### 3.4. Downregulation of Mcl-1 Interferes with *S. aureus*-Induced Cytoprotection

**Figure 4:**
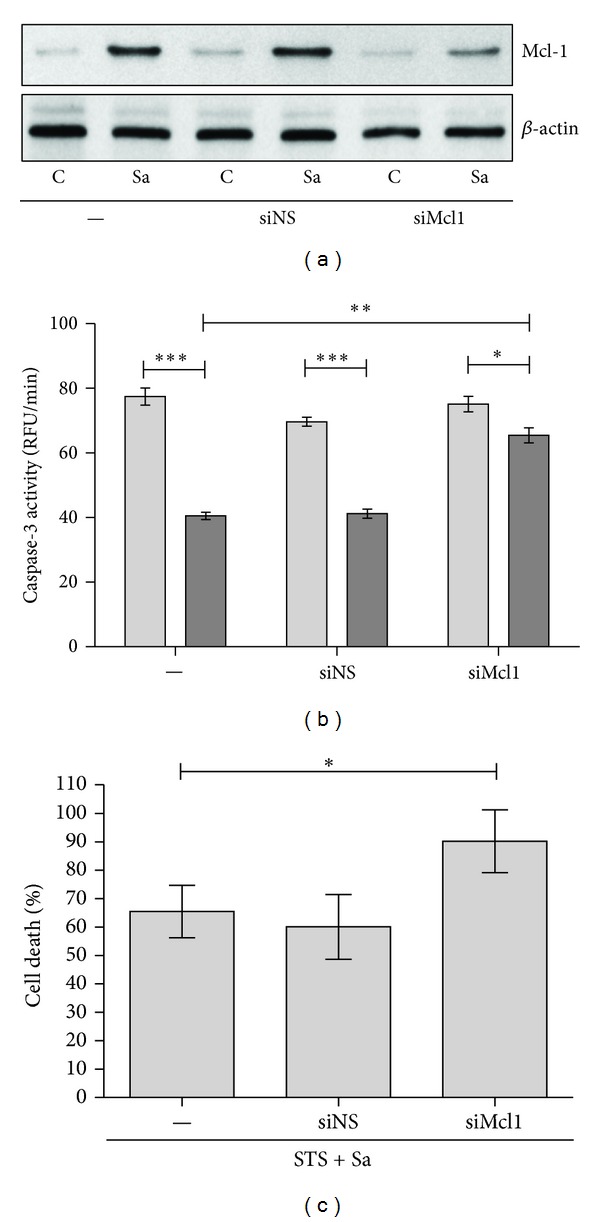
Effect of Mcl-1 expression on cytoprotection induced by *S. aureus* in hMDMs. (a) Human macrophages were treated with an *MCL1 *siRNA (siMCL1) or a nonspecific siRNA (siNS). At 24 h following transfection macrophages were infected with *S. aureus* at an MOI 1 : 50. After additional 24 h cells were collected and Mcl-1 expression was detected by immunoblot. Data are representative of three separate experiments using hMDMs derived from different donors. (b) The increase in caspase-3 activity (RFU/min) induced by STS in *MCL1* knockdown macrophages infected with *S. aureus*. Twenty-four hours after treatment with siRNA, hMDMs were infected with *S. aureus* (24 h), followed by treatment with STS at a concentration of 1 *μ*M for 18 h. The measurement of caspase-3 activity (RFU/min) in cell lysates was performed using DEVD-AFC as a substrate. The figure is representative of three experiments, using hMDMs cultures obtained from different donors. Light bars—STS, dark bars—Sa + STS. Data represent means ± SD. *P < 0.05; **P < 0.01; ***P < 0.001. (c) Increased susceptibility to the cytotoxic effects of staurosporine (STS) in *MCL1* knockdown macrophages infected with *S. aureus*. Twenty-four hours after treatment with siRNA, hMDMs were infected with *S. aureus* (24 h), followed by treatment with STS at a concentration of 1 *μ*M for 24 h. Plasma membrane permeabilisation or cell lysis induced in the hMDMs was assessed by measuring LDH activity in the culture medium. LDH activity in the media of cells treated only with STS was arbitrarily set as 100%. Results were calculated based on data (± SD) from three separate experiments. *P < 0.05.

As we described previously *S. aureus* protects infected macrophages, both human and murine, against induced cell death [7]. Thus, to further determine whether the survival of infected macrophages in response to proapoptotic stimulants was dependent on Mcl-1 synthesis, RNA interference with siRNA was used to selectively silence *MCL1* gene expression. Treatment of cells with an *MCL1*-specific siRNA, but not a nonspecific control siRNA, resulted in specific and efficient suppression of Mcl-1 protein levels at 24–72 h after-transfection (data not shown) without effect on macrophages viability. The infection of macrophages 24 h after-transfection has not influenced the low level of already silenced protein within 24–48 h after infection (Figure 4(a)). The increasing caspase-3 activity (Figure 4(b)) and a lactate dehydrogenase leaking from macrophages (Figure 4(c)) revealed that the knockdown of *MCL1* expression significantly attenuated the *S. aureus*-exerted cytoprotection of cells in a staurosporine-induced cell death model. Our data also indicates (Supplementary Figure 2) that silencing of *Mcl-1* in *S. aureus*-infected macrophages partly ablates the cytoprotection against spontaneous cell death. This observation confirmed that Mcl-1 plays an important role in preventing apoptosis in *S. aureus*-infected human macrophages. 

### 3.5. *S. aureus*-Dependent Mcl-1 Expression Is Regulated by IL-6

**Figure 5:**
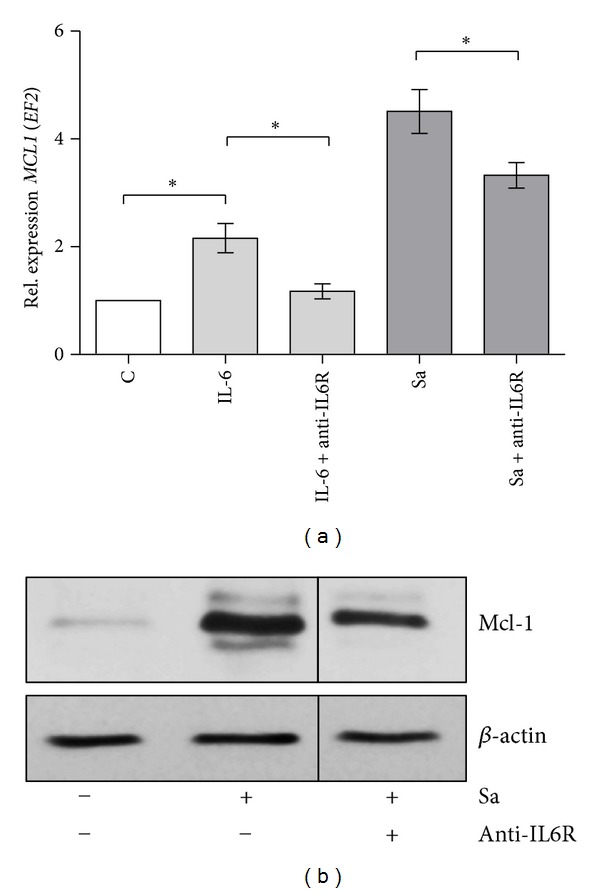
Mcl-1 expression induced by *S. aureus* is mediated by IL-6. (a) hMDMs were preincubated with anti-IL6 receptor antibodies (1 *μ*g/mL) for 30 min and then infected with *S. aureus* at an MOI of 1 : 50 and/or stimulated with IL-6 (200 ng/mL). At 7 h p.i., RNA was extracted and relative *MCL1* expression was measured by qRT-PCR. Diagram shows the mean values calculated from the results of at least three independent real-time reactions using hMDMs derived from different donors. Bars represent mean relative expression ± SD. *P < 0.05. (b) hMDMs were preincubated with anti-IL6 receptor (1 *μ*g/mL) antibodies for 30 min and then infected with *S. aureus* Newman at an MOI of 1 : 50. The effect of *S. aureus* on Mcl-1 protein synthesis was measured 20 h after-infection by immunoblot. Shown is a representative immunoblot from three separate experiments performed on hMDMs derived from different donors.

IL-6 upregulates Mcl-1 in human myeloma cells [18]. To determine whether IL-6 was also playing a role in *S. aureus*-induced Mcl-1 expression in hMDMs, macrophages were infected with *S. aureus, *what leads to IL-6 secretion within 24 h after infection (Supplement Figure 3). Both high mRNA and protein Mcl-1 expression observed after bacteria phagocytosis (Figures 5(a) and 5(b), resp.) was markedly reduced upon treatment of cells with IL-6 receptor (R) neutralising antibodies. Together, these results demonstrated that *S. aureus*-induced Mcl-1 expression is partly mediated by IL-6.

### 3.6. The NF*κ*B Pathway Is Involved in IL-6-Dependent Regulation of Mcl-1 Expression Induced by *S. aureus*

**Figure 6:**
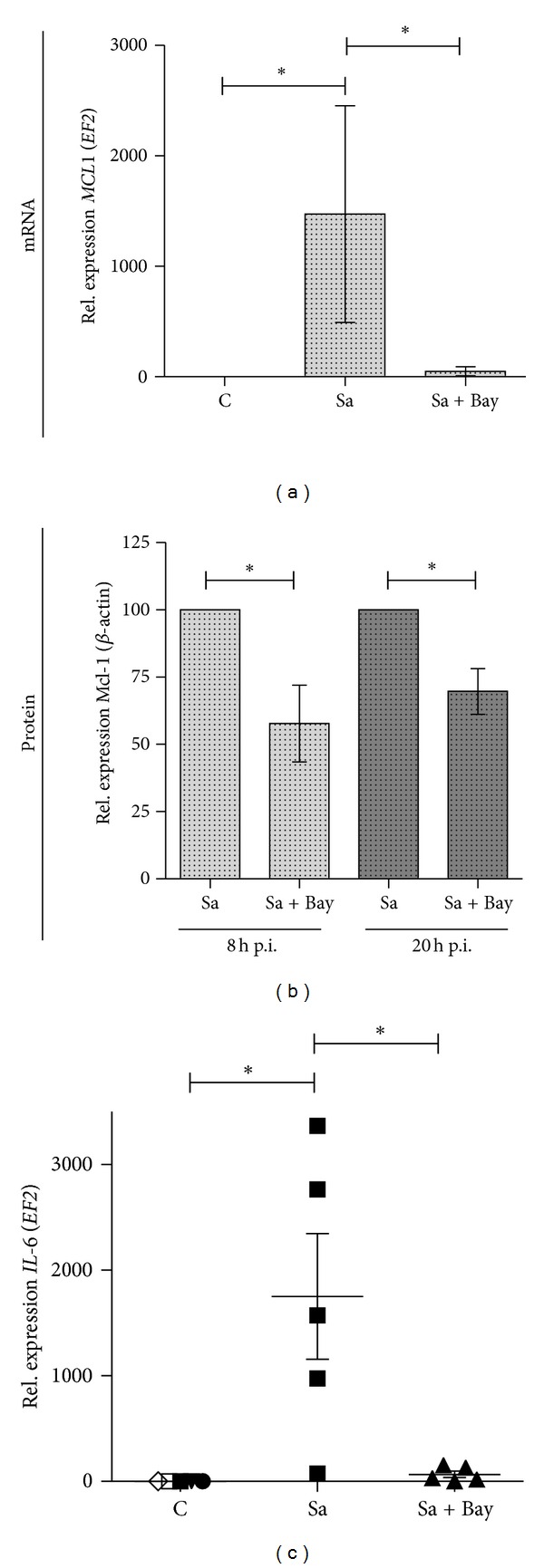
NF*κ*B is required for Mcl-1 expression induced by *S. aureus. *(a, b) hMDMs were pretreated for 30 min with Bay 11-7095 (40 *μ*M) followed by *S. aureus* infection at an MOI of 50. After the indicated times, RNA and protein were extracted and Mcl-1 expression levels were determined by qRT-PCR and immunoblot (a and b, resp.). Data represent the means ± SD from three separate experiments. *P < 0.05. (c) The effect of NF*κ*B inhibition on IL-6 expression was measured by qRT-PCR. Shown are the mean values calculated from the results of five independent real-time reactions using hMDMs derived from different donors. Bars represent mean relative expression ± SD; *P < 0.05.

A variety of intracellular signalling pathways are activated by pathogens. Among them, activation of NF*κ*B has been shown to be critical for cytoprotection of infected cells [19]. Moreover, *S. aureus* is a potent inducer of NF*κ*B activity as was confirmed in infected macrophages by EMSA (Supplement Figure 4). To determine the effect of inhibition of the NF*κ*B pathway on Mcl-1 expression in hMDMs, macrophages were infected with *S. aureus *followed by incubation for 6 h with a specific NF*κ*B-inhibitor, and then the levels of *MCL1* were assessed in comparison to untreated infected cells. As seen in Figure 6(a), inhibition of the NF*κ*B pathway abrogated the *S. aureus*-induced increase in *MCL1* gene transcription. This effect was confirmed at the protein level as well (Figure 6(b)). These results strongly suggested that Mcl-1 expression in *S. aureus*-infected cells is dependent on NF*κ*B. Since IL-6 transcription is upregulated in an NF*κ*B-dependent manner [20], we investigated the possibility that NF*κ*B stimulated *MCL1* expression indirectly, via IL-6. *S. aureus*-induced production of IL-6 in infected macrophages was abrogated by treatment with Bay 11-7095, the NF*κ*B-specific cell-permeable inhibitor, which indicated that IL-6 production was absolutely dependent on NF*κ*B (Figure 6(c)). Taken together, these findings indicate that IL-6-mediated Mcl-1 expression in infected macrophages is regulated through the NF*κ*B pathway. 

Since we proved that the *S. aureus* mediated Mcl-1 induction in both human and murine model, therefore, in all further experiments we routinely use the hMDMs and/or RAW 264.7 cell line.

### 3.7. NF*κ*B and IL-6 Are Required for *S. aureus*-Induced Inhibition of Macrophage Apoptosis

**Figure 7:**
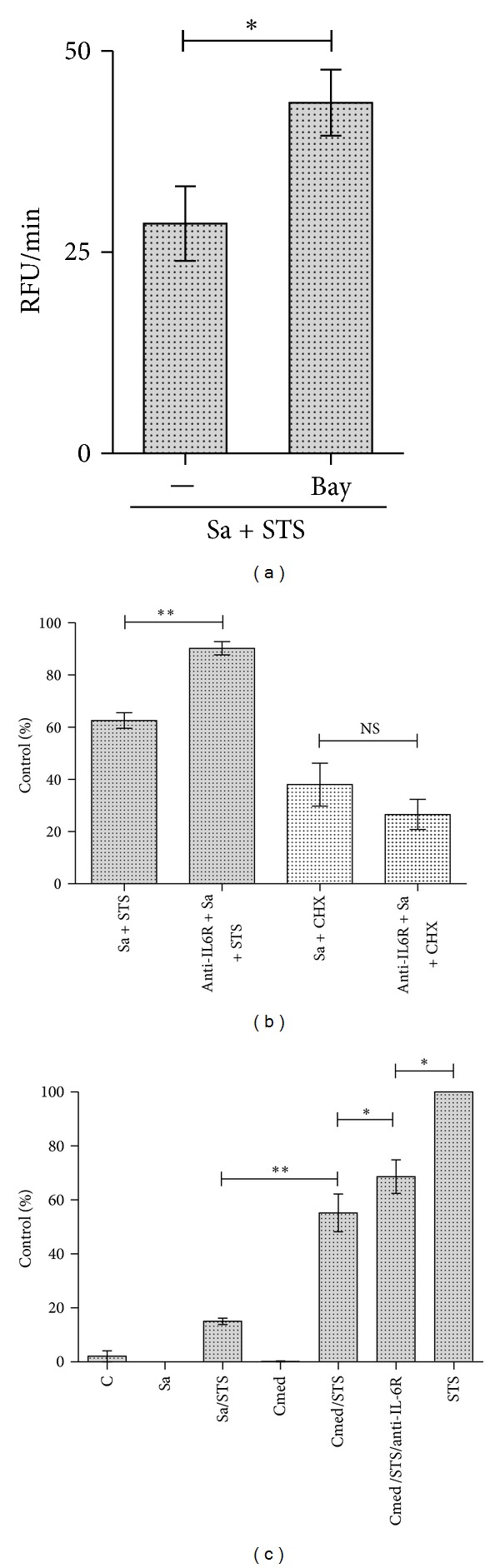
The role of NF*κ*B and IL-6 in cytoprotection induced by *S. aureus. *(a) Inhibition of *S. aureus*-induced cytoprotection upon treatment with an NF*κ*B inhibitor was assessed by measuring caspase-3 activation. RAW 264.7 macrophages were pretreated for 30 min with Bay 11-7095 (4 *μ*M) followed by bacterial infection at an MOI of 5 for 2 h. STS was added for an additional 3 h, and then caspase-3 activity was measured in cell lysates (as described in Section 2). Data represent mean ± SD caspase-3 activity (RFU/min) from three separate experiments. *P < 0.05. (b, c) The influence of IL-6 on STS- (1 *μ*M) or CHX- (10 *μ*M) induced cytotoxicity in *S. aureus-*infected macrophages. (b) hMDMs were preincubated with anti-IL6 receptor antibodies (1 *μ*g/mL) for 1 h followed by *S. aureus* infection at an MOI of 1 : 50 for 2. STS or CHX was added for 6 h, and then permeabilisation of the plasma membrane or cell lysis was determined by measuring LDH activity in conditioned media. Cell death after treatment with STS or CHX alone was set as 100%. Diagram shows the mean values calculated from the results of three independent experiments using hMDMs derived from different donors. Bars represent mean relative expression ± SD; **P < 0.01; NS: not significant. (c) RAW 264.7 cells were infected with *S. aureus* (MOI 1 : 5) or stimulated with conditioned medium collected from infected RAW 264.7 cells 5 h p.i. alone (cmed) or with anti-IL6 receptor (1 *μ*g/mL) antibodies (cmed + anty-IL6R). At 2 h after stimulation, macrophages were treated with STS for another 4 h and then caspase-3 activity was measured. Caspase-3 activation induced by STS alone was set as 100%. Shown are mean values calculated from the results of three independent experiments. Bars represent mean relative expression ± SD; *P < 0.05; **P < 0.01.

Based on the fact that *S. aureus* delayed apoptosis through Mcl-1 upregulation in infected macrophages we investigated whether NF*κ*B-dependent pathways and/or IL-6 were necessary for *S. aureus*-induced inhibition of apoptosis. In this experiment we used both hMDMs and murine macrophage RAW 264.7 cell line, the latter to verify the results of Mcl-1 induction in mice joints by *S. aureus* infection. Preincubation of macrophages with an NF*κ*B inhibitor (Bay 11-7095) reversed antiapoptotic effect (measured by caspase-3 activation) induced by *S. aureus *in staurosporine-treated cells (Figure 7(a)). The viability of hMDMs was also assessed after blocking IL-6 signalling. Pretreatment of human macrophages with IL-6R neutralising antibodies partly abolished *S. aureus*-induced protection against cell death exerted by staurosporine, but had no effect on cytoprotection against cycloheximide- (CHX-) mediated cell death (Figure 7(b)). To determine whether IL-6 acted in an autocrine manner to prevent cell death induced by staurosporine, conditioned media from *S. aureus*-infected macrophages containing abundant IL-6 (Supplement Figure 3) was added to noninfected macrophages, followed by treatment of the cells with staurosporine. To confirm the role of secreted upon *S. aureus* infection IL-6 on suppression of macrophages apoptosis we blocked the action of IL-6 using anti-IL6 receptor (1 *μ*g/mL) antibodies. Measurement of caspase-3 activity revealed a significant effect, albeit one that was weaker than that induced by *S. aureus* infection, of the conditioned medium on cytoprotection, which can be partly reversed by IL-6 signalling inhibition (Figure 7(c)). Cumulatively, these data demonstrate that the induction of IL-6 production in macrophages upon *S. aureus* infection is an important factor in the activation of downstream events that lead to suppression of the intrinsic apoptotic pathway through *de novo *synthesis Mcl-1. 

## 4. Discussion

Results from recent *in vitro* studies have demonstrated that macrophages infected with *S. aureus *are resistant to apoptosis (both induced and spontaneous), and this apparent cytoprotective effect is a consequence of the significant upregulation of antiapoptotic genes, especially those involved in the mitochondrial pathway, such as *MCL1* [7]. Here, we investigated this phenomenon in more detail and showed for the first time that *S. aureus*-induced resistance to cell death is highly dependent on Mcl-1 expression since specific silencing of *MCL1* abrogated the effect. In this respect, *S. aureus* clearly resembles *M. tuberculosis* or respiratory syncytial virus (RSV), well-known obligatory intracellular pathogens that employ a similar strategy [21, 22]. 

Mcl-1 is a short-lived cytoplasmic protein (half-life of approximately 3 h) destined for prompt degradation by the proteasome [23, 24]. This fast proteolytic removal of Mcl-1 was markedly slowed in *S. aureus*-infected macrophages. The level of Mcl-1 rapidly increased within 2 h after bacterial infection, reached a maximum at 6 h, and then remained elevated for up to 24 h. In addition to enhanced *MCL1* gene expression [7], this increase in Mcl-1 levels was due in large part to increased Mcl-1 stability. In contrast to control cells, the relative levels of Mcl-1 in infected cells were only slightly decreased by inhibition of protein translation. This is consistent with the resistance of *S. aureus* infected macrophages to cell death experimentally induced by cycloheximide. Thus, apoptosis inhibition in macrophages following *S. aureus* infection involves both enhancement of *MCL1* transcription and the prolonged life-time of Mcl-1 in infected cells. This underscores the essential role of antiapoptotic Mcl-1 in *S. aureus*-induced cytoprotection in macrophages and argues that hijacking of Mcl-1 function is essential for survival of infected cells thus facilitating survival of intracellular invaders. 

The confirmation of Mcl-1 role in staphylococcal infection was obtained studying *S. aureus*-induced septic arthritis. Our data clearly shows enhanced Mcl-1 expression in infected joints. This suggests that also *in vivo S. aureus* yields cytoprotection to infected cells establishing a safe haven impervious to attack by antibacterial forces of the immune system. Taking into account that the majority of synovial cells in the cartilage-synovium junction that participate in the destructive process are macrophages, the prolonged life-spam of infected phagocytes may be directly linked to the severity of septic arthritis lesions. Our hypothesis is corroborated by both the level of bacterial infection/spreading and the Mcl-1 expression in peritoneal macrophages isolated from *S. aureus*-infected mice, which are higher in comparison to macrophages from infected animals additionally exposed to NF*κ*B or IL-6 signalling inhibitors (data not shown). 

IL-6 plays an important role in pathogenesis of septic arthritis promoting synovitis, manifested by stimulation of chemokines and adhesion molecules and infiltration of inflammatory cells, such as macrophages and lymphocytes. IL-6 has been also shown to be essential for Mcl-1 expression thus promoting accumulation of macrophages [25]. Therefore, *S. aureus*-induced IL-6 secretion could be responsible for Mcl-1 upregulation in infected macrophages. In keeping with this, Mcl-1 induction was partly attenuated by specifically blocking the IL-6R, which suggests an autocrine role of IL-6 in the *S. aureus*-induced cytoprotection of infected macrophages through increased expression of Mcl-1. This effect is regulated by the NF*κ*B pathway as it is apparent from the observation that Mcl-1 expression was decreased upon inhibition of NF*κ*B. However, it should be underlined that, since Mcl-1 can be induced by several different survival and differentiation signals, both on transcriptional and posttranscriptional levels, the involvement of NF*κ*B and IL-6 pathways in this process is a part of the very complex regulation network.

Interactions between hosts and pathogens vary depending on the strategies employed by the pathogens to deter host immunity. Therefore, defining the defence mechanisms that are selective for a particular pathogen or, conversely, the precise virulence strategy of the pathogen is a crucial step in predicting the outcome of infection. Described here and in a previous report [7] sustained Mcl-1 expression and cytoprotection of macrophages by *S. aureus *following infection appears to be specific for this bacterium, since engulfment of Gram-negative bacteria or latex beads yielded negligible effects. Such highly specific activation of Mcl-1 expression could be beneficial for the host or advantageous for *S. aureus*. Pathogen persistence or replication in a protected intracellular environment and dissemination before infected cells are removed by Fas-mediated apoptosis argue strongly for the latter option. A similar strategy is employed by *M. tuberculosis*, an obligatory intracellular pathogen, which employs a strategy whereby intracellular replication is prolonged by Mcl-1 upregulation and inhibition of apoptosis, hence promoting chronic persistence [22]. The opposite mode of action has been documented for *Streptococcus pneumoniae* infection, where initial enhancement of macrophage viability allows professional phagocytes to eradicate the infection [26]. 

Therefore, based on presented data we propose the scenario in which intracellular *S. aureus *makes macrophages resistant to apoptosis thus using these mobile cells as the Trojan horse to disseminate. The follow-up study aimed to unambiguously verify this attractive hypothesis is in progress in our laboratory.

## 5. Conclusions

To sum up, our work describes the mechanism of Mcl-1 induction and its pivotal role in *S. aureus*-mediated cytoprotection of infected macrophages. Moreover, the upregulation of Mcl-1 *in vivo* indicates that observed phenomenon plays a role during staphylococcal infection. 

## Supplementary Material

Supplementary Figure1. Clinical and bacteriological examination of *S. aureus* induced septic arthritis.
Supplementary Figure 2. Increased susceptibility to the spontaneous cell death in *MCL1* knockdown macrophages infected with *S. aureus*.
Supplementary Figure 3. IL-6 secretion induced by *S. aureus* in hMDMs.
Supplementary Figure 4. NF*κ*B activity induced by *S. aureus* in hMDMs.

## Figures and Tables

**Table 1:** Oligonucleotide sequences used in qRT-PCR.

Oligonucleotide	Annealing (°C)	Sequence
EF-2 F	62	5′-GACATCACCAAGGGTGTGCAG-3′
EF-2 R	62	5′-TCAGCACACTGGCATAGAGGC-3′
IL-6 F	54	5′-CATCTTTGGAAGGTTCAGGTTTGT-3′
IL-6 R	54	5′-AGCCCTGAGAAAGGAGACATGTA-3′
MCL1 F	62	5′-TAAGGACAAAACGGGACTGG-3′
MCL1 R	62	5′-ACCAGCTCCTACTCCAGCAA-3′
MCL1S F	62	5′-GCAACCACGAGACGGCC-3′
MCL1S R	62	5′-GATGCCACCTTCTAGGTCCTCTAC-3′
