# Supplementary material for: The Role of Mcl-1 in S. aureus-Induced Cytoprotection of Infected Macrophages
Source: Mediators Inflamm. 2013 Jan 28;2013:427021. doi: 10.1155/2013/427021 (PMC3569898; doi:10.1155/2013/427021)
Supplement: Supplementary file 1 — Supplementary Figure1. Clinical and bacteriological examination of S. aureus induced septic arthritis. Supplementary Figure 2. Increased susceptibility to the spontaneous cell death in MCL1 knockdown macrophages infected with S. aureus. Supplementary Figure 3. IL-6 secretion induced by S. aureus in hMDMs. Supplementary Figure 4. NFκB activity induced by S. aureus in hMDMs. [file 427021.f1.zip › 427021.f1/description.pdf]

The bacteriological, clinical and molecular examination of S. aureus-*Induced Arthritis in mice model.*

S. aureus is the causative agent in about 60% of nongonococcal bacterial arthritis cases, a disease characterized among others by robust influx of macrophages and their sustain activation in joints [1, 2]. Therefore, we determined Mcl-1 expression in inflamed joints in the previously established murine model of *S. aureus* arthritis [3]. For this purpose the low mortality dose of i.v. injected *S. aureus* was established (5×10^7 CFU) using DBA1 mice model (Supplementary Figure 1(a)). At day 8 after injection all animals showed clear symptoms of arthritis (Supplementary Figure 1(b)). Bacteriological examination of joints, spleen, and kidneys revealed the abundant 5 load of *S. aureus* in 100% of mice (Supplementary Figure 1(c)). This finding correlates with inflammatory response manifested by IL-6 secretion (Supplementary Figure 1(d)).

Downregulation of Mcl-1 Interferes with S. aureus- *Induced Cytoprotection.*

As we described previously *S. aureus* protects infected macrophages, both human and murine, against induced cell death [4]. Our results revealed that the knockdown of *MCL1* expression significantly attenuated the *S. aureus*-exerted cytoprotection of cells in a staurosporine-induced cell death model. Furthermore, we confirm the role of *S. aureus* induced Mcl-1 against spontaneous cell death. Since human monocyte-derived macrophages (hMDMs) are primary cells in which we have rarely observed any symptoms of spontaneous cell death up to 20 days post differentiation, therefore, among all performed experiments using siRNA against *Mcl-1* we observed the process of spontaneous cell death only in case of one blood/cells donor. In that case the silencing of *Mcl-1* in *S. aureus* infected macrophages partly ablates the cytoprotection (Supplementary Figure 2). This observation confirmed that Mcl-1 plays an important role in preventing apoptosis in *S. aureus*-infected human macrophages. Moreover, slight induction of cell death sporadically observed in mock-infected cells was always inhibited in *S. aureus*-infected cells.

S. aureus *induces IL-6 secretion in infected hMDMs.*

IL-6 upregulates Mcl-1 in human myeloma cells [5]. To determine whether IL-6 was also playing a role in *S. aureus* induced Mcl-1 expression in hMDMs, firstly we established the secretion of IL-6 by *S. aureus* infected macrophages. Our data indicates that *S. aureus* leads to IL-6 secretion within 24 h after infection (Supplement Figure 3). Together, these results suggest that *S. aureus*-induced Mcl-1 expression can be mediated by IL-6.

The NFκB Pathway Is Involved in IL-6-Dependent Regulation of Mcl-1 Expression Induced by S. aureus .

A variety of intracellular signalling pathways are activated by pathogens. Among them, activation of NF*κ*B has been shown to be critical for cytoprotection of infected cells [6]. Therefore, we established the activation of NF*κ*B in *S. aureus* infected macrophages by EMSA (Supplement Figure 4).

References

1. Goldenberg DL, Chisholm PL, Rice PA: Experimental models of bacterial arthritis: a microbiologic and histopathologic characterization of the arthritis after the intraarticular injections of Neisseria gonorrhoeae, Staphylococcus aureus, group A streptococci, and Escherichia coli. J Rheumatol 1983;10:5-11.

2. Goldenberg DL: Infectious arthritis complicating rheumatoid arthritis and other chronic rheumatic disorders. Arthritis Rheum 1989;32:496-502.

3. Bremell T, Lange S, Yacoub A, Ryden C, Tarkowski A: Experimental Staphylococcus aureus arthritis in mice. Infect Immun 1991;59:2615-2623.

4. Koziel J, Maciag-Gudowska A, Mikolajczyk T, Bzowska M, Sturdevant DE, Whitney AR, Shaw LN, DeLeo FR, Potempa J: Phagocytosis of Staphylococcus aureus by macrophages exerts cytoprotective effects manifested by the upregulation of antiapoptotic factors. PLoS One 2009;4:e5210.

5. Puthier D, Bataille R, Amiot M: IL-6 up-regulates mcl-1 in human myeloma cells through JAK / STAT rather than ras / MAP kinase pathway. Eur J Immunol 1999;29:3945-3950.

6. Bonizzi G, Karin M: The two NF-kappaB activation pathways and their role in innate and adaptive immunity. Trends Immunol 2004;25:280-288.
