# Supplementary material for: The Role of Mcl-1 in S. aureus-Induced Cytoprotection of Infected Macrophages
Source: Mediators Inflamm. 2013 Jan 28;2013:427021. doi: 10.1155/2013/427021 (PMC3569898; doi:10.1155/2013/427021)
Supplement: Supplementary file 1 — Supplementary Figure1. Clinical and bacteriological examination of S. aureus induced septic arthritis. Supplementary Figure 2. Increased susceptibility to the spontaneous cell death in MCL1 knockdown macrophages infected with S. aureus. Supplementary Figure 3. IL-6 secretion induced by S. aureus in hMDMs. Supplementary Figure 4. NFκB activity induced by S. aureus in hMDMs. [file 427021.f1.zip › 427021.f1/mat.427021.v2.pptx]

## Slide 1
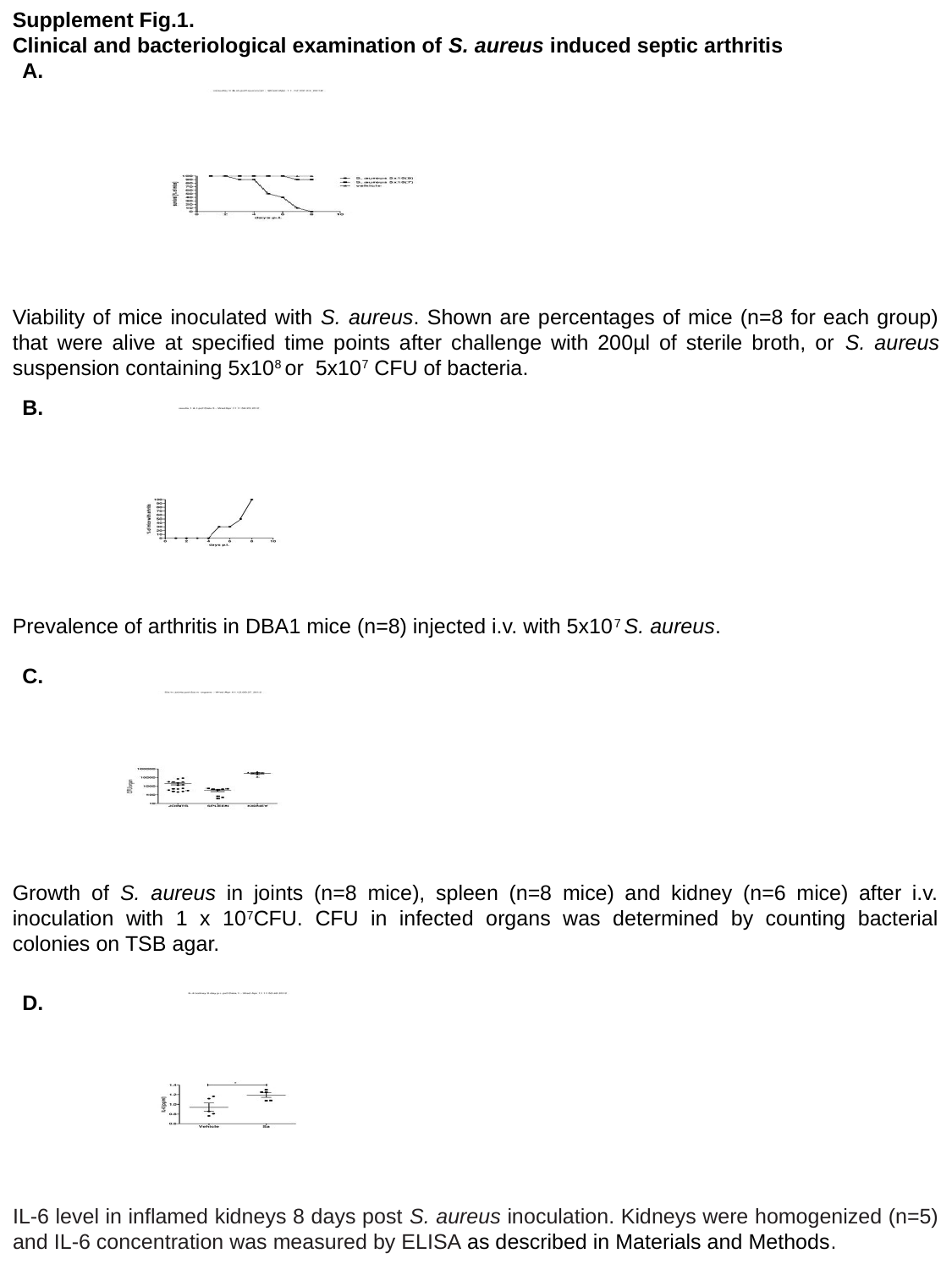

Supplement Fig.1.
Clinical and bacteriological examination of S. aureus induced septic arthritis
A.
Viability of mice inoculated with S. aureus. Shown are percentages of mice (n=8 for each group) that were alive at specified time points after challenge with 200µl of sterile broth, or S. aureus suspension containing 5x10^8^ or 5x10^7^ CFU of bacteria.
B.
Prevalence of arthritis in DBA1 mice (n=8) injected i.v. with 5x10^7^ S. aureus.
C.
Growth of S. aureus in joints (n=8 mice), spleen (n=8 mice) and kidney (n=6 mice) after i.v. inoculation with 1 x 10^7^CFU. CFU in infected organs was determined by counting bacterial colonies on TSB agar.
D.
IL-6 level in inflamed kidneys 8 days post S. aureus inoculation. Kidneys were homogenized (n=5) and IL-6 concentration was measured by ELISA as described in Materials and Methods.

## Slide 2
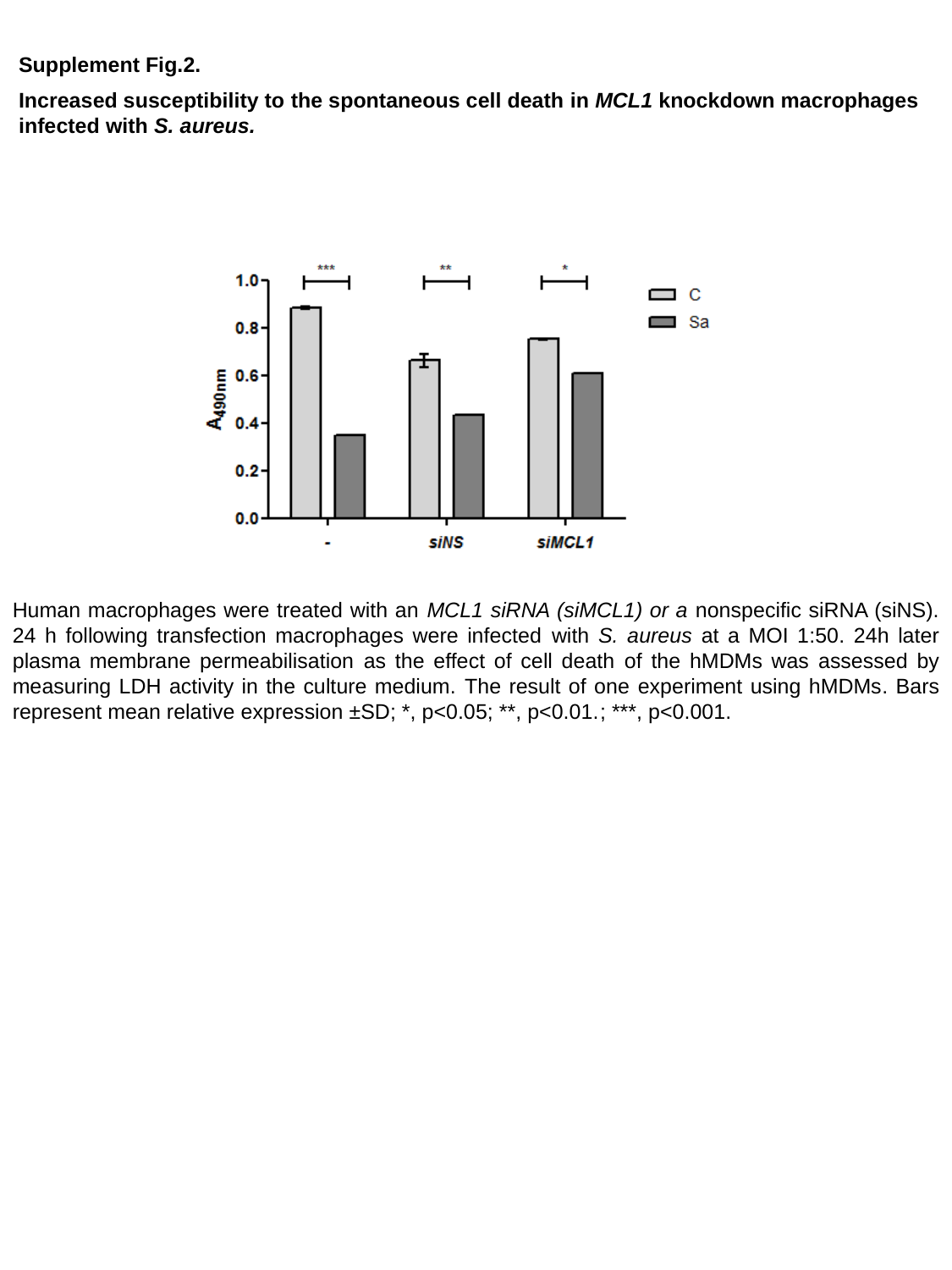

Supplement Fig.2.
Increased susceptibility to the spontaneous cell death in MCL1 knockdown macrophages infected with S. aureus.
Human macrophages were treated with an MCL1 siRNA (siMCL1) or a nonspecific siRNA (siNS). 24 h following transfection macrophages were infected with S. aureus at a MOI 1:50. 24h later plasma membrane permeabilisation as the effect of cell death of the hMDMs was assessed by measuring LDH activity in the culture medium. The result of one experiment using hMDMs. Bars represent mean relative expression ±SD; *, p<0.05; **, p<0.01.; ***, p<0.001.

## Slide 3
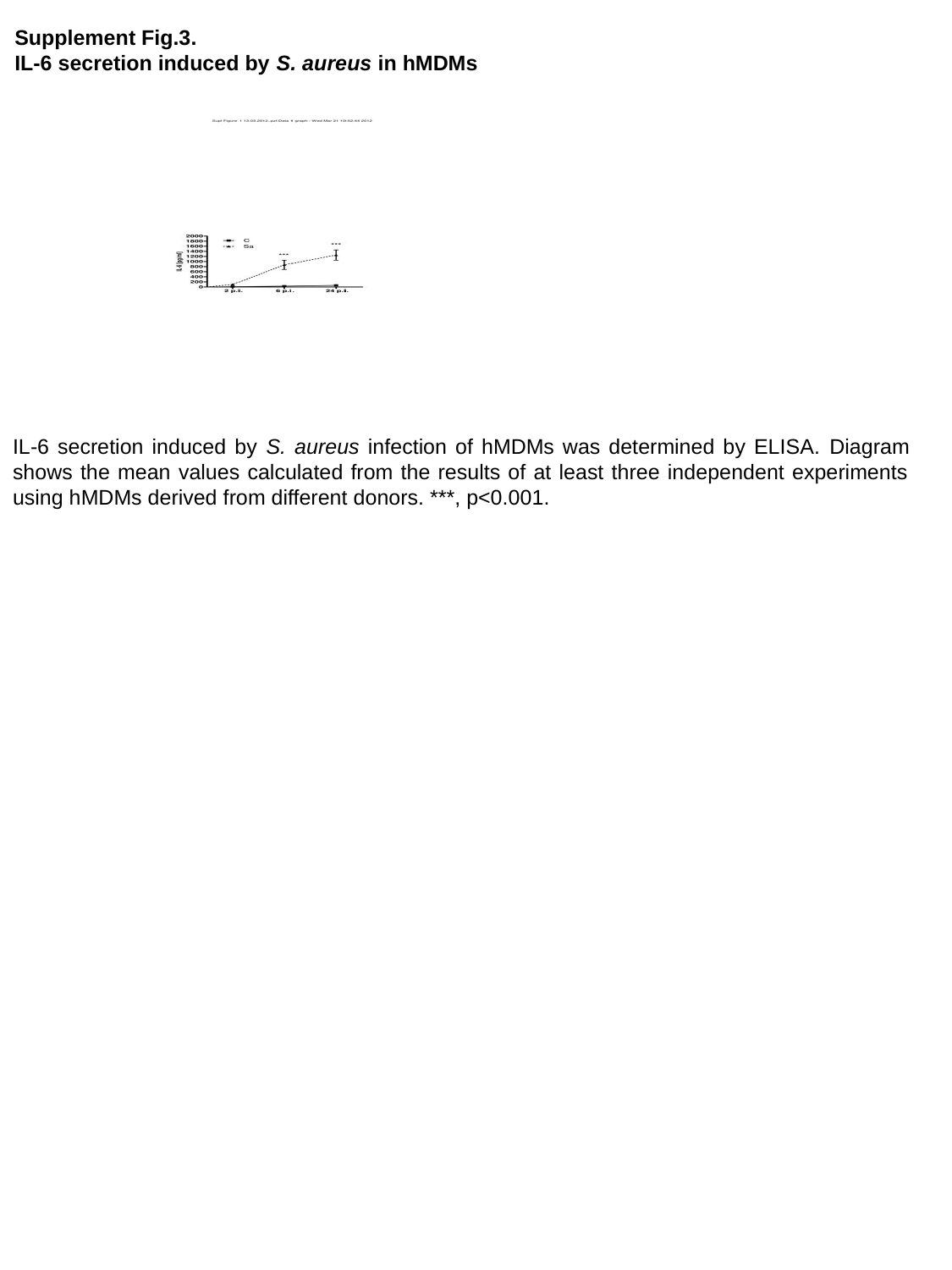

Supplement Fig.3.
IL-6 secretion induced by S. aureus in hMDMs
IL-6 secretion induced by S. aureus infection of hMDMs was determined by ELISA. Diagram shows the mean values calculated from the results of at least three independent experiments using hMDMs derived from different donors. ***, p<0.001.

## Slide 4
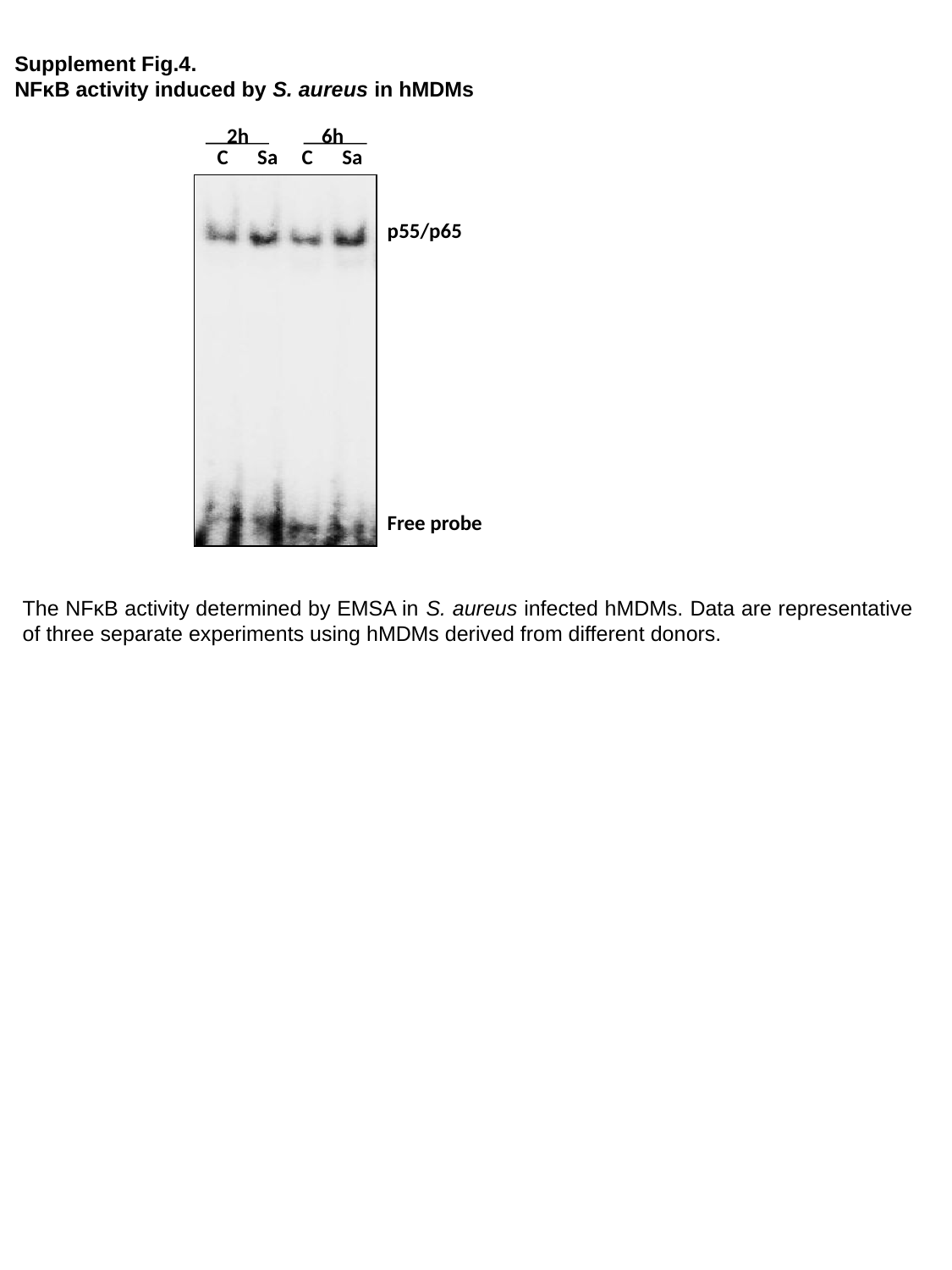

Supplement Fig.4.
NFκB activity induced by S. aureus in hMDMs
 2h 6h
 C Sa C Sa
p55/p65
Free probe
The NFκB activity determined by EMSA in S. aureus infected hMDMs. Data are representative of three separate experiments using hMDMs derived from different donors.

## Slide 5
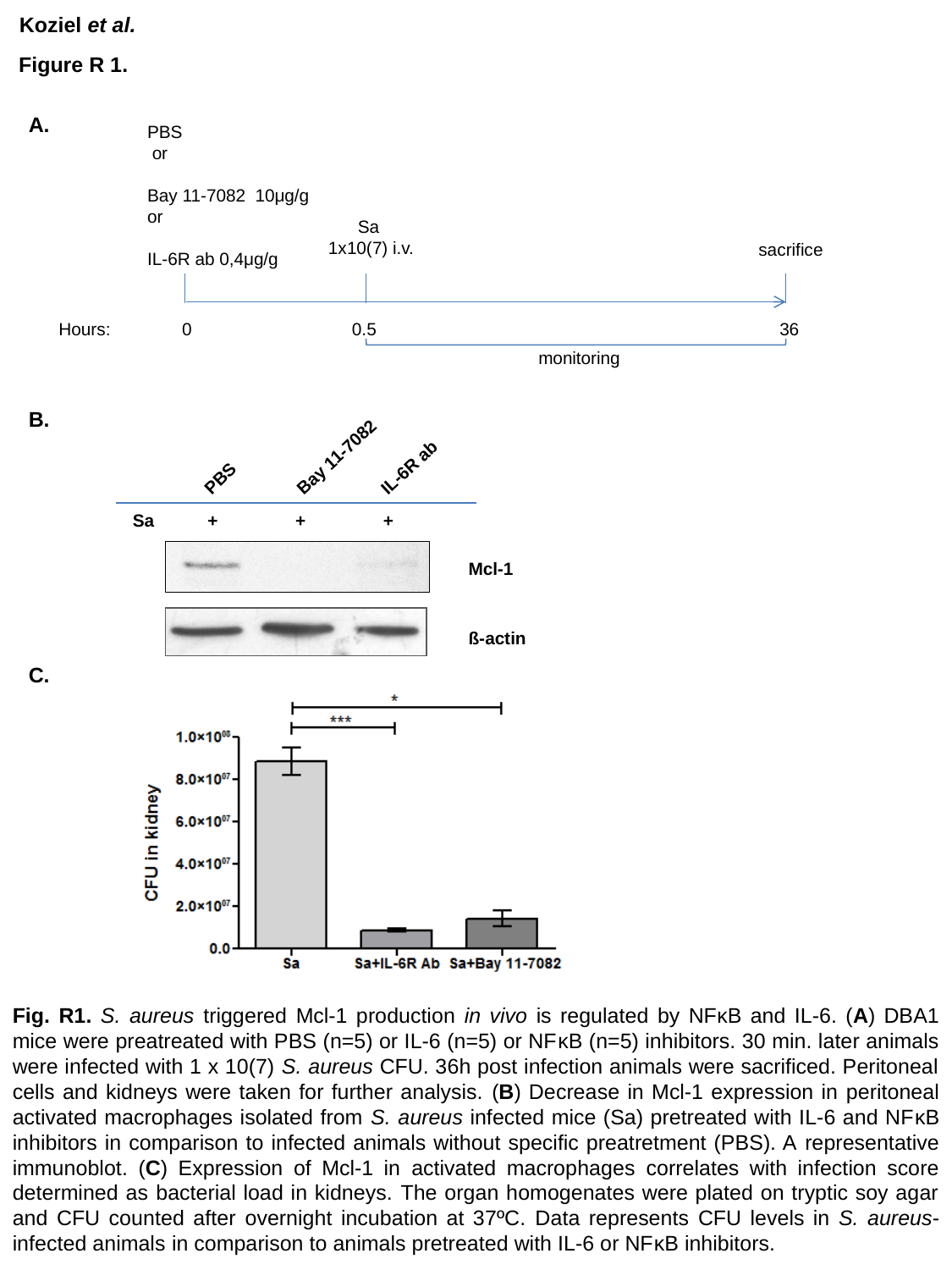

Koziel et al.
Figure R 1.
A.
PBS
 or
Bay 11-7082 10μg/g or
IL-6R ab 0,4μg/g
Sa
1x10(7) i.v.
sacrifice
 0 0.5 36
Hours:
monitoring
B.
IL-6R ab
Bay 11-7082
PBS
Sa + + +
Mcl-1
ß-actin
C.
Fig. R1. S. aureus triggered Mcl-1 production in vivo is regulated by NFκB and IL-6. (A) DBA1 mice were preatreated with PBS (n=5) or IL-6 (n=5) or NFκB (n=5) inhibitors. 30 min. later animals were infected with 1 x 10(7) S. aureus CFU. 36h post infection animals were sacrificed. Peritoneal cells and kidneys were taken for further analysis. (B) Decrease in Mcl-1 expression in peritoneal activated macrophages isolated from S. aureus infected mice (Sa) pretreated with IL-6 and NFκB inhibitors in comparison to infected animals without specific preatretment (PBS). A representative immunoblot. (C) Expression of Mcl-1 in activated macrophages correlates with infection score determined as bacterial load in kidneys. The organ homogenates were plated on tryptic soy agar and CFU counted after overnight incubation at 37ºC. Data represents CFU levels in S. aureus-infected animals in comparison to animals pretreated with IL-6 or NFκB inhibitors.

## Slide 6
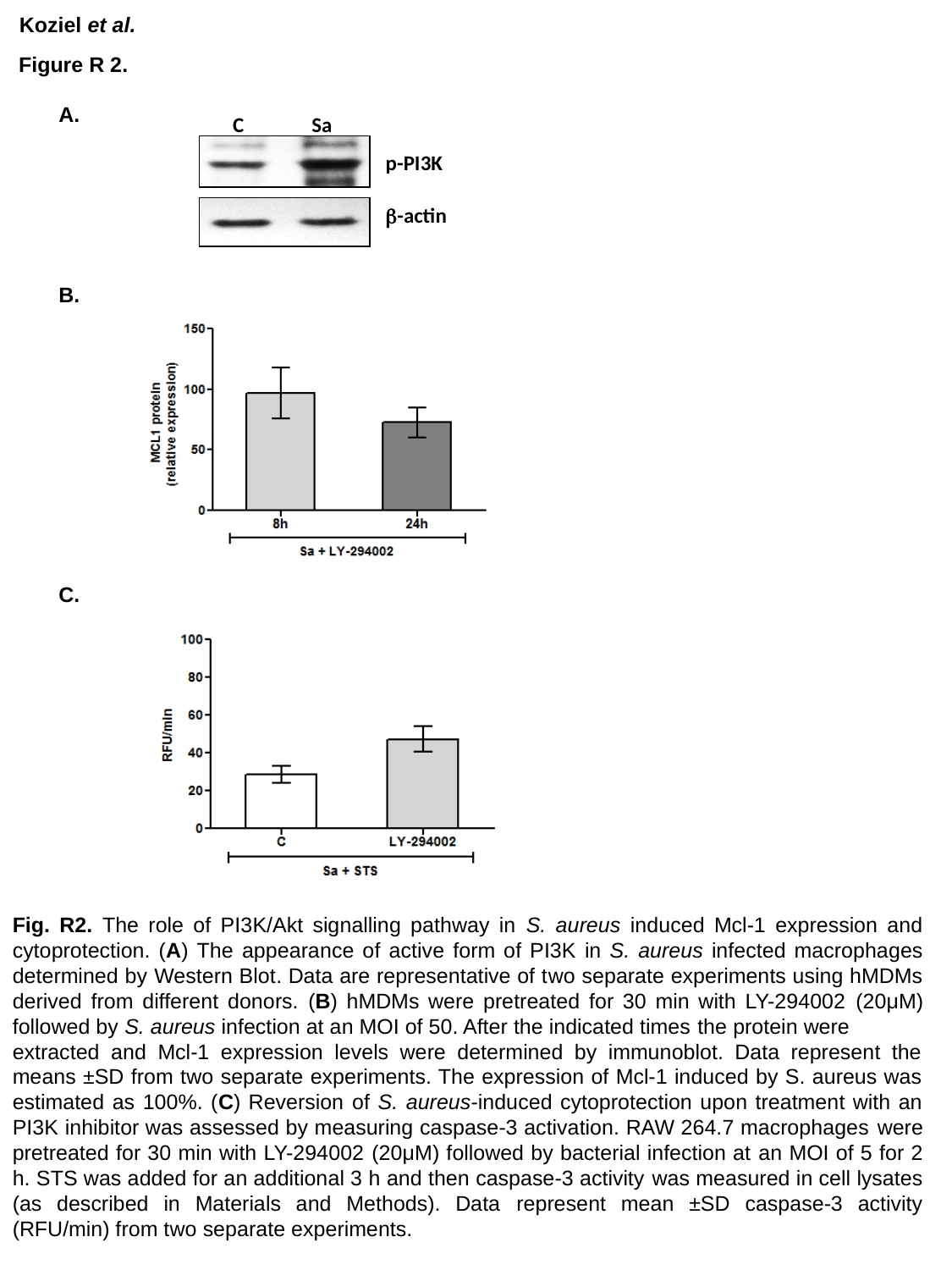

Koziel et al.
Figure R 2.
A.
 C Sa
 p-PI3K
 b-actin
B.
C.
Fig. R2. The role of PI3K/Akt signalling pathway in S. aureus induced Mcl-1 expression and cytoprotection. (A) The appearance of active form of PI3K in S. aureus infected macrophages determined by Western Blot. Data are representative of two separate experiments using hMDMs derived from different donors. (B) hMDMs were pretreated for 30 min with LY-294002 (20μM) followed by S. aureus infection at an MOI of 50. After the indicated times the protein were
extracted and Mcl-1 expression levels were determined by immunoblot. Data represent the means ±SD from two separate experiments. The expression of Mcl-1 induced by S. aureus was estimated as 100%. (C) Reversion of S. aureus-induced cytoprotection upon treatment with an PI3K inhibitor was assessed by measuring caspase-3 activation. RAW 264.7 macrophages were pretreated for 30 min with LY-294002 (20μM) followed by bacterial infection at an MOI of 5 for 2 h. STS was added for an additional 3 h and then caspase-3 activity was measured in cell lysates (as described in Materials and Methods). Data represent mean ±SD caspase-3 activity (RFU/min) from two separate experiments.
